# GAS6/Axl Signaling Modulates Blood-Brain Barrier Function Following Intravenous Thrombolysis in Acute Ischemic Stroke

**DOI:** 10.3389/fimmu.2021.742359

**Published:** 2021-10-18

**Authors:** Zhen-Ni Guo, Jie Liu, Junlei Chang, Peng Zhang, Hang Jin, Xin Sun, Yi Yang

**Affiliations:** ^1^ Stroke Center, Department of Neurology, The First Hospital of Jilin University, Changchun, China; ^2^ Clinical Trial and Research Center for Stroke, Department of Neurology, The First Hospital of Jilin University, Changchun, China; ^3^ Center for Protein and Cell-based Drugs, Institute of Biomedicine and Biotechnology, Shenzhen Institute of Advanced Technology, Chinese Academy of Sciences, Shenzhen, China

**Keywords:** acute ischemic stroke, blood-brain barrier, hemorrhagic transformation, recombinant tissue plasminogen activator, growth arrest-specific 6, Axl

## Abstract

**Background and Purpose:**

Recent studies have shown that several proteins, including Axl, are related to hemorrhagic transformation (HT) following intravenous thrombolysis by affecting blood-brain barrier (BBB) function. However, the effects of these proteins on BBB function have been studied primarily in animal models. In this study, we aimed to identify serum protein markers that predict HT following intravenous thrombolysis in patients with acute ischemic stroke (AIS) and verify whether these serum proteins regulate BBB function and HT in animal stroke models.

**Methods:**

First, 118 AIS patients were enrolled in this study, including 52 HT patients and 66 non-HT patients. In Step 1, baseline serum levels of Axl, angiopoietin-like 4, C-reactive protein, ferritin, hypoxia-inducible factor-1 alpha, HTRA2, Lipocalin2, matrix metallopeptidase 9, platelet-derived growth factor-BB, and tumor necrosis factor alpha were measured using a quantitative cytokine chip. Next, sequence mutations and variations in genes encoding the differentially expressed proteins identified in Step 1 and subsequent function-related proteins were detected. Finally, we verified whether manipulation of differentially expressed proteins affected BBB function and HT in a hyperglycemia-induced rat stroke model.

**Results:**

Serum Axl levels were significantly lower in the HT group than in the non-HT group; none of the other protein markers differed significantly between the two groups. Genetic testing revealed that sequence variations of *GAS6* (the gene encoding the Axl ligand)-derived long non-coding RNA, *GAS6-AS1*, were significantly correlated with an increased risk of HT after intravenous thrombolysis. In animal studies, administration of recombinant GAS6 significantly reduced brain infarction and neurological deficits and attenuated BBB disruption and HT.

**Conclusions:**

Lower serum Axl levels, which may result from sequence variations in *GAS6-AS1*, are correlated with an increased risk of HT after intravenous thrombolysis in stroke patients. Activation of the Axl signaling pathway by the GAS6 protein may serve as a therapeutic strategy to reduce HT in AIS patients.

## Introduction

Stroke is a major cause of disability and death worldwide (1, 2). Recombinant tissue plasminogen activator (rt-PA) is currently the most effective treatment for acute ischemic stroke (AIS) (3). However, hemorrhagic transformation (HT), a major complication of rt-PA therapy, increases life-threatening risk and contributes to adverse events in patients with AIS (4, 5). The mechanism of HT after thrombolysis is complicated, and disruption of the blood-brain barrier (BBB) plays an important role in this process (6, 7). Clinical studies of BBB permeability imaging demonstrate that increased BBB permeability predicts incidences of HT in patients with AIS (8, 9). Animal studies have resulted in similar conclusions (10, 11).

Previous studies have reported several proteins associated with BBB function, including Axl (12), angiopoietin-like 4 (ANGPTL4) (13), C-reactive protein (CRP) (14), ferritin (15, 16), hypoxia-inducible factor-1 alpha (HIF-1α) (17), HTRA2 (18), lipocalin2 (19), matrix metallopeptidase 9 (MMP-9) (20), platelet-derived growth factor-BB (PDGF-BB) (21, 22), and tumor necrosis factor alpha (TNF-α) (23). However, the effects of these proteins on BBB function have been studied primarily in animal or *in vitro* models, and their roles in BBB disruption and HT after intravenous thrombolysis in patients with AIS are largely unknown.

Thus, in the present study, we sought to (1) investigate baseline serum levels of the above ten proteins in cases of AIS with and without HT who underwent rt-PA therapy; (2) detect sequence mutations and variations in the genes encoding differentially expressed proteins identified in Step 1 and subsequent function-related proteins; and (3) verify whether modulation of the differentially expressed proteins in Step 1 can improve BBB function and reduce HT in animal stroke models.

## Methods

The clinical study design was approved by the Ethics Committee of the First Hospital of Jilin University, and all participants provided written informed consent. The animal study design was approved by the Animals Ethics Committee of Jilin University, and all procedures were performed in accordance with the Guide for the Care and Use of Laboratory Animals.

### Study Design

Step 1: Detection of baseline serum levels of Axl, ANGPTL4, CRP, ferritin, HIF-1α, HTRA2, lipocalin2, MMP-9, PDGF-BB, and TNF-α in patients with AIS with and without HT using a quantitative cytokine chip.

Step 2: Detection of gene sequence variations of genes and related genes of the differentially expressed proteins identified in Step 1.

Step 3: Verification of whether the manipulation of the differentially expressed proteins in Step 1 affected BBB function and HT in animal stroke models.

### Clinical Study

#### Participants

We conducted a retrospective study of patients diagnosed with ischemic stroke who underwent standard rt-PA treatment (0.9 mg/kg) at the Department of Neurology in the First Hospital of Jilin University between June 2016 and June 2018. Additional enrollment criteria included willingness to undergo venous blood sample collection and computed tomography scans at the time of admission and 24 h after thrombolysis. We excluded patients with other neurological disorders, myocardial infarction or unstable angina within six months, as well as patients with atrial fibrillation. With these enrollment criteria, a total of 224 AIS patients were initially included in this study. For the HT group, 52 consecutive patients who experienced HT and met the criteria above were included. Sixty-six non-HT patients who met the criteria above and did not experience HT during the same period were selected from 172 eligible patients. Computer-based randomization was used to select a group of non-HT patients from 172 eligible patients.

#### Definition of HT

HT was defined as any newly developed intracranial hemorrhage detected by computed tomography 24 h after rt-PA that was not detected by computed tomography prior to thrombolysis (24).

#### Human Cytokine Antibody Array

We analyzed cytokines using the Quantibody Human custom array (RayBiotech, Norcross, GA), which detects and quantifies ten cytokines simultaneously (25–27). In short, monoclonal antibodies complimentary to numerous proteins were printed on glass slides to bind to the corresponding proteins in serum. Then, the slides were incubated with a biotinylated secondary antibody mixture and detected with Cy3-labeled streptavidin. Each analyte was measured in quadruplicate. A laser scanner (InnoScan 300 Microarray Scanner, Innoscansys, France) was then used to scan the slides. The RayBiotech analysis tool was used to analyze the signal values.

#### Blood Collection and DNA Extraction

Before rt-PA treatment, venous blood samples were collected from the basal vein of each patients. Samples were centrifuged (3000 rpm, 10 min) at 4°C; then, leukocytes were rapidly frozen and stored at -80°C until analysis. Genomic DNA of the isolated leukocytes was extracted using the Blood DNA Kit (Cat. #GO-HYAS, GeneOn BioTech, China) according to the manufacturer’s instructions.

#### Detection of Gene Mutation and Polymorphism

At the genetic level, we tested *Axl, GAS6* (the gene encoding the ligand for Axl), and the *GAS6*-derived long non-coding RNA (*GAS6-AS1*) (28), including 20 SNPs of *Axl*, 1 InDel of *Axl*, 15 SNPs of* GAS6*, and 19 SNPs of *GAS6-AS1*. The detailed protocols regarding target sequencing, data pre-processing, variant discovery, annotation, and statistical analysis, followed previously established methodology (29).

##### Target Sequencing

Qualified genomic DNA of each individual was hybridized with the designed target capture array to enrich exonic DNA in each library. Then, we performed sequencing with 150 bp pair reads independently for each captured library on the Illumina Xten platform to ensure that the average coverage of each sample was approximately 700-2600 fold.

##### Data Pre-Processing

Samples were aligned to the NCBI human genome reference assembly (hg19) using the Burrows-Wheeler Aligner. Next, we employed Picard Mark Duplicates to label the duplicate reads to reduce biases caused by data generation (such as PCR amplification). Using the Genome Analysis Toolkit (GATK v3.3), the BAM files were processed to realign around known indels. We then recalibrated the base quality scores for the individual base calls in each sequence read.

##### Variant Discovery

Germline short variant discovery proceeds from analysis-ready BAM files and produces variant calls. GATK (v3.3) HaplotypeCaller was used to call variants per sample in targeted and flanking regions for each individual in order to produce a file in GVCF format. We then performed joint genotyping to combine the multisample GVCF. Next, we performed GenotypeGVCFs to obtain a multisample genotype for allsites. Finally, the hard-filter was applied to produce the final multisample callset with the desired balance of precision and sensitivity.

##### Annotation

SnpEff was used to separate single nucleotide variants into different functional categories according to their genic location and their expected effect on encoded gene products, based on information from the RefSeq database. All variants were further annotated by the control population of the 1000 Genomes Project (2014 Oct release, http://www.1000genomes.org), ExAC (http://exac.broadinstitute.org), EVS (http://evs.gs.washington.edu/EVS), Disease databases of ClinVar (http://www.ncbi.nlm.nih.gov/clinvar), and OMIM (http://www.omim.org). In addition, we categorized the single nucleotide variants into known or novel groups according to whether they were present in the Single Nucleotide Polymorphism database (version 150).

##### Statistical Analysis

After sample quality controls, 52 samples from HT patients and 66 samples from non-HT patients in targeted sequencing were employed for statistical analysis. After unqualified variants (–biallelic-only –geno 0.2 –hwe 0.0001) were filtered, single-variant association analysis for single nucleotide variants and inDel was performed by case-control association analysis with the Fisher’s model using PLINK 1.9.

### Animal Experiment

Rats were housed in a 12-h light/dark cycle, with room temperature maintained at 23°C. Food and water were freely available. A total of 184 male Sprague-Dawley rats weighing 250–280 g (Changsheng Inc., Liaoning, China) were included in this study. The sample size was determined based on that of previous studies evaluating the effects of Axl in middle cerebral artery occlusion (MCAO) or intracerebral hemorrhage (ICH) animal models (30, 31) and previous results obtained in our laboratory. The expected sample size of each group was approximately 6. The exclusion criteria were as follows: dying before the chosen endpoint or without a deficit after 1 h of reperfusion except in case of sham animals. Our experiment was divided into two parts to determine the effect of rGAS6 on hyperglycemia-induced rats with HT at 24 and 72 h after MCAO, respectively. In the first part, 108 rats were weighed and numbered according to their weight. Computer-based randomization was used to divide the animals into three groups, namely the sham group, sham+dextrose+PBS (n=36); the HT group, MCAO+dextrose+PBS (n=36); and the recombinant GAS6 (rGAS6) group, MCAO+dextrose+rGAS6 (Axl agonist, n=36). A total of 16 rats that did not meet the inclusion criteria were replaced immediately. A total of 54 rats were included in the second part. They were randomly divided into three groups, the sham group (n=18); the HT group (n=18); and the rGAS6 group (n=18). A total of 6 rats that did not meet the inclusion criteria were replaced immediately. The individuals performing the experiments were blinded to both group membership and outcome.

#### Hyperglycemia-Induced HT Rat Models

Rats were intraperitoneally injected with 50% dextrose (6 mL/kg) 15 min before MCAO to induce acute hyperglycemia. Anesthesia was induced by 4% isoflurane in a nitrous oxide/oxygen mixture (70/30) and maintained by 1.5% isoflurane using a facemask. A feedback-controlled heating pad was used to maintain rectal temperature at 37.0°C during and after surgery. MCAO was induced according to previously established methodology (32, 33). Briefly, the right external carotid artery was dissected through a midline neck incision, and its branches were also dissected and coagulated. Next, the external carotid artery was cut, leaving a stump as long as possible to attach to the common carotid artery. A 4-0 nylon suture (2636-A5, Beijing Xi Nong Technology Co Ltd., China) was inserted through the stump and advanced into the internal carotid artery approximately 19-20 mm to occlude the origin of the middle cerebral artery. 90 min after occlusion, the animals were re-anesthetized, and the suture was removed. Rats in the sham group underwent the same procedures without the suture insertion.

#### Intranasal Administration of rGAS6

The rats were re-anesthetized and treated with rGAS6 60 min after occlusion. Nasal administration was performed according to previously established methodology (34). PBS or rGAS6 dissolved in PBS (20 μg/kg, 0.25 µg/µl, R&D system, Minneapolis, MN) was delivered alternately into the bilateral nares, one drop every 2 min.

#### Neurological Score

Neurobehavioral defects were evaluated by a blinded investigator using a modified Garcia neurological score 24 and 72 h after MCAO (35). The modified Garcia score contained 6 items including spontaneous activity, limb symmetry, forepaw outstretching, body proprioception, response to vibrissae touch, and climbing. The scores of spontaneous activity, limb symmetry, and forepaw outstretching were scored from 0~3, while the body proprioception, response to vibrissae touch, and climbing were scored from 1~3. The total score ranged from 3 (most severe deficit) to 18 (maximum).

#### Assessment of Cerebral Infarct Volume

Rats were anesthetized and perfused with heparinized saline *via* the ascending aorta 24 and 72 h after MCAO. The brains were immediately removed and sliced into 2.0-mm thick coronal sections. Each slice was immersed in 2% 2,3,5-triphenyltetrazolium chloride in the dark at 37°C for 20 min. Then, the non-infarcted area was stained red, while the infarcted area remained white. We used ImageJ software (National Institutes of Health, NIH, USA) to measure the infarct areas by tracing around the white area in each section. The infarct volume was expressed as a percentage of the whole contralateral hemisphere (36).

#### Brain Water Content

At 24 h after MCAO, animals were anesthetized and decapitated as previously reported (37). Their brains were quickly removed, and the water on the surface of the cerebral tissue was dried with filter paper. The brains were then dissected into the following three sections: the cerebellum, and left and right hemispheres. Each sample was weighed on an electric analytic balance to acquire the wet weight and then dried at 100°C for 24 h to acquire the dry weight. Brain water content was calculated as (wet weight–dry weight)×100/wet weight.

#### Spectrophotometric Assay of Hemoglobin

Cerebral hemorrhage was quantified using a spectrophotometric assay of brain hemoglobin content (38). Rats were anesthetized and perfused with heparinized saline *via* the ascending aorta 24 and 72 h after MCAO. The brain was quickly removed and dissected into the left and right hemispheres. The two parts were then homogenized in 0.1 mol/L of PBS and centrifuged (13000 g, 30 min) at 4°C. A “virtual” model of hemorrhage was used to derive a standard curve. Briefly, incremental volumes of homologous blood (0, 2, 4, 8, 16, and 32 μL) were added to the perfused naïve brain. After homogenization and centrifugation, Drabkin reagent (1.6 mL; Sigma) was added to 0.4 mL aliquots of supernatant, and a spectrophotometer (Spectronix 3000, Milton-Roy, Rochester, NY) was used to measure the optical density at 540 nm. The content of total hemispheric hemoglobin was compared with the standard curve to obtain hemorrhage volume (μL).

#### Measurement of BBB Function

Evans blue extravasation was measured to evaluate BBB function in each group. In total, 4 mL/kg of 2% Evans blue dye in 0.9% saline was injected *via* tail vein 24 h after reperfusion. Two hours after injection, rats were anesthetized and sacrificed by transcardial perfusion to completely remove the intravascular localized dye. After decapitation, brains were rapidly removed and divided into left and right hemispheres. Each hemisphere was weighed and then placed in 3 mL of 50% trichloroacetic acid solution, homogenized, incubated, and centrifuged (12000 rpm, 30 min). A spectrophotometer (Ultrospec 3; Pharmacia LKB) was used to measure the absorbance of the supernatant at 610 nm. To derive the standard curve, incremental weights of Evans blue (0, 0.25, 0.5, 1, 1.5, 2 and 2.5 μg) were added to 3 mL of 50% trichloroacetic acid solution, respectively. Then, a spectrophotometer was used to measure the optical density at 610 nm. The Evans blue content was presented as micrograms per gram of brain tissue compared with the standard curve (39).

#### Western Blots

Western blots were performed according to previously established methodology (40). The primary antibodies used were rabbit monoclonal anti-Axl (Abcam, Cambridge, MA; used for total Axl) and rabbit polyclonal anti-phosphorylation-Axl (Bioss Inc., Beijing, China). Rabbit polyclonal anti-β-actin and the secondary antibodies were all from Beijing Biosynthesis Biotechnology. Immunoreactivity was detected using an ECL Plus chemiluminescence reagent kit (Amersham Biosciences, Arlington Heights, IL).

#### Statistical Analysis

Analyses were performed using the Statistical Program for Social Sciences version 17.0 (SPSS, IBM, West Grove, PA, USA). Measurement data are presented as mean ± standard deviation or median (interquartile range) depending on the data distribution pattern. The Mann-Whitney test or Student’s t-test was used to analyze statistical significances between the two independent groups. Logistic regression model was used to explore the association between multiple protein indexes and hemorrhagic transformation. Proteins were converted into binary variables (high and low) according to its median. Variables with P<0.1 in univariate analyses were included in the multiple analysis as confounding factors. One-way analysis of variance or the Kruskal-Willis H test was used to compare the differences between multiple groups (in the animal study section). The post-comparison analyses used were the Tukey and Bonferroni tests. Count data are expressed as absolute values and percentages and were identified using a chi-square test. Calculated two-tailed P­-values<0.05 were considered to be statistically significant.

## Results

### Clinical Study

#### Participant Characteristics

This study included 118 patients who underwent rt-PA therapy, including 52 HT patients (17 men, age: 63.27 ± 11.59) and 66 non-HT patients (18 men, age: 61.76 ± 10.97). The demographic and clinical characteristics of the included patients are presented in **Table 1**.

**Table 1:** Demographical and clinical features of HT and non-HT groups.

	HT group (n = 52)	Non-HT group (n = 66)	*P*-value
Sex (male/female)	35/17	48/18	0.52
Age (years)	63.27 ± 11.59	61.76 ± 10.97	0.47
Hypertension, n (%)	32 (61.5)	36 (54.5)	0.45
Diabetes, n (%)	15 (28.8)	11 (16.7)	0.11
Hyperlipidemia, n (%)	4 (7.7)	15 (22.7)	0.027
Atrial fibrillation, n (%)	11 (21.2)	9 (13.6)	0.28
History of stroke, n (%)	8 (15.4)	4 (6.1)	0.096
Application of antiplatelet agents, n (%)	8 (15.4)	7 (10.6)	0.44
Systolic blood pressure, mmHg	152.00 ± 18.84	147.80 ± 20.66	0.26
Diastolic blood pressure, mmHg	89.21 ± 11.79	87.23 ± 10.95	0.35
Blood glucose, mmol/L	8.10 (6.80, 10.58)	7.05 (6.00, 8.55)	0.005
Platelet, L	203.37 ± 54.82	198.41 ± 58.56	0.64
Low density lipoprotein cholesterol, mmol/L	2.84 ± 0.78	2.86 ± 0.74	0.88
NIHSS, points	12.0 (8.0, 15.0)	11.0 (6.75, 13.0)	0.29

HT, hemorrhagic transformation; NIHSS, National Institute of Health stroke scale.

#### Serum Proteins in HT and Non-HT Groups

**Figure 1:**
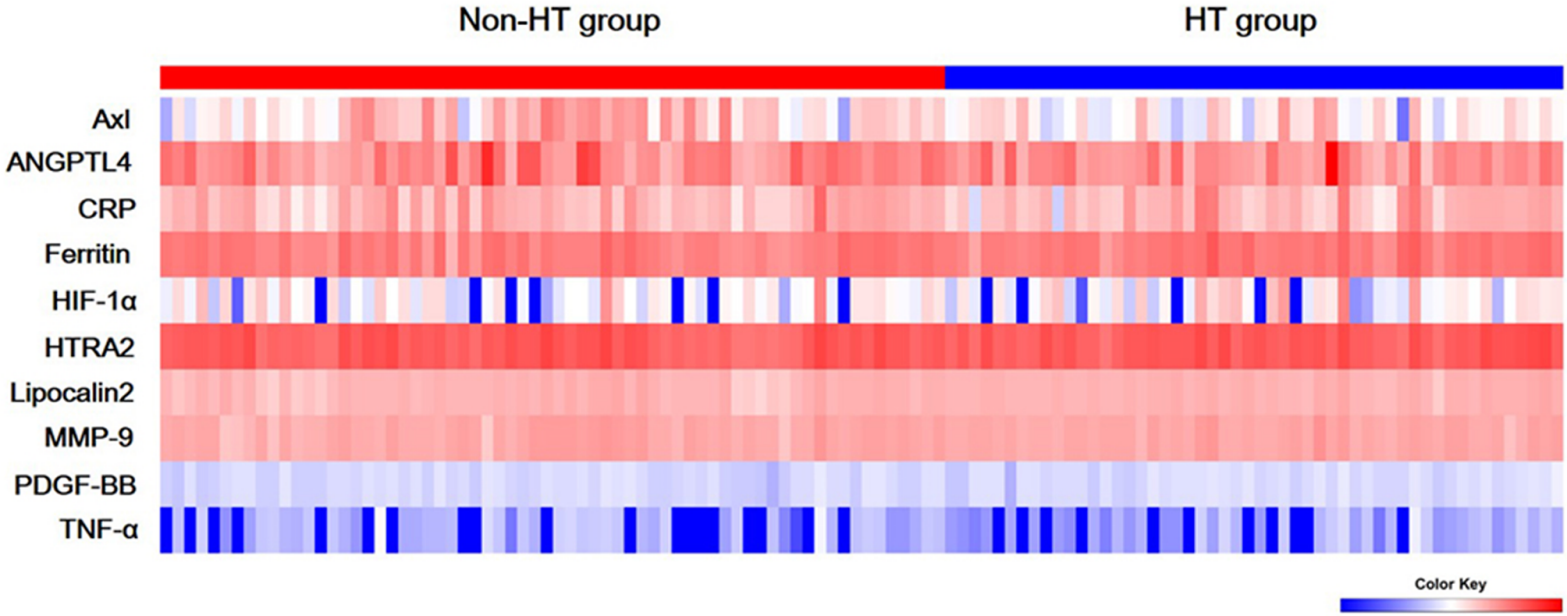
Serum levels of ten biomarkers. Heat map of quantitative protein chip of Axl, angiopoietin-like 4 (ANGPTL4), C-reactive protein (CRP), ferritin, hypoxia-inducible factor-1 alpha (HIF-1α), HTRA2, Lipocalin2, Matrix metallopeptidase 9 (MMP-9), platelet-derived growth factor-BB (PDGF-BB), and tumor necrosis factor alpha (TNF-α) in acute ischemic stroke (AIS) with/without hemorrhagic transformation (HT).

The comparison of Axl, ANGPTL4, CRP, ferritin, HIF-1α, HTRA2, lipocalin2, MMP-9, PDGF-BB, and TNF-α levels revealed that Axl levels were significantly lower in the HT group compared with the non-HT group (*P*<0.001) (**Figure 1** and **Table 2**). The risk of hemorrhagic transformation in patients with high Axl is 0.177 times that of patients with low Axl after adjusting for hyperlipidemia, history of stroke, and blood glucose (**Table 3**).

**Table 2:** Results of the Human Cytokine Antibody Array.

Indicators (pg/ml)	HT group (n = 52)	Non-HT group (n = 66)	Z	*P*
Axl	1219.25 (727.58, 2813.67)	3521.98 (1329.12, 9401.39)	-3.94	<0.001
ANGPTL4	15692.84 (10671.96, 23447.44)	18364.70 (10805.05, 30421.49)	-1.43	0.15
CRP	5587.28 (3687.05, 8209.64)	6203.38 (3716.98, 9383.56)	-0.66	0.51
Ferritin	30121.05 (25609.18, 33738.50)	26704.34 (20492.54, 34090.38)	-1.66	0.097
HIF-1a	1008.47 (279.33, 2344.25)	836.67 (361.52, 2068.20)	-0.32	0.75
HTRA2	70069.17 ± 20754.44	65660.58 ± 22452.86	-1.19	0.24
Lipocalin2	6671.72 (6207.20, 7174.41)	6431.45 (5665.88, 7247.73)	-1.66	0.097
MMP-9	9035.50 ± 1726.44	8900.01 ± 2145.92	-0.070	0.94
PDGF-BB	322.46 ± 76.17	298.46 ± 72.57	-1.86	0.063
TNF-a	70.15 (29.20, 122.00)	102.43 (0, 182.27)	-0.71	0.48

HT, hemorrhagic transformation; ANGPTL4, angiopoietin-like 4; CRP, C-reactive protein; HIF-1α, hypoxia-inducible factor-1 alpha; MMP-9, matrix metallopeptidase; PDGF-BB, platelet-derived growth factor-BB; TNF-α, tumor necrosis factor alpha.

**Table 3:** The Association between Multiple Protein Indexes and HT.

	OR (95% CI)^*^	P
Axl (high *vs*. low)	0.177 (0.074-0.426)	<0.001
ANGPTL4 (high *vs*. low)	0.687 (0.318-1.488)	0.342
CRP (high *vs*. low)	1.052 (0.482-2.298)	0.899
Ferritin (high *vs*. low)	2.394 (1.068-5.368)	0.034
HIF-1a (high *vs*. low)	1.073 (0.493-2.338)	0.859
HTRA2 (high *vs*. low)	1.637 (0.747-3.590)	0.218
Lipocalin2 (high *vs*. low)	2.254 (1.007-5.045)	0.048
MMP-9 (high *vs*. low)	1.252 (0.569-2.755)	0.577
PDGF-BB (high *vs*. low)	2.273 (1.019-5.068)	0.045
TNF-a (high *vs*. low)	0.588 (0.269-1.282)	0.182

^*^Adjusted for hyperlipidemia, history of stroke, and blood glucose.

#### Genetic Results

At the genetic level, we tested the *Axl*, *GAS6* (the gene encoding the ligand for Axl), and *GAS6-AS1* (28), including 20 SNPs of *Axl*, 1 InDel of *Axl*, 15 SNPs of *GAS6*, and 19 SNPs of *GAS6-AS1*. Our results revealed that the *GAS6-AS1* SNPs (rs1803628, rs9604573, and rs7140110) were highly related to an increased risk of HT after intravenous thrombolysis (*P*=0.010, 0.017, 0.025, respectively; **Table 4**). The gene sequence variations of *Axl* or *GAS6* were not correlated with HT following intravenous thrombolysis.

**Table 4:** Association of GAS6 gene mutations and polymorphisms with hemorrhagic transformation.

Gene	SNP ID	Base Change	Variant Type	Allele Frequency		OR	*P*
				Ctrl (%)	HT (%)		
GAS6-AS1	rs1803628	G>A	Intron variant	7.69	19.23	2.86	0.010
GAS6-AS1	rs9604573	G>A	Intron variant	16.15	29.81	2.20	0.017
GAS6-AS1	rs7140110	T>C	Intron variant	16.15	28.85	2.10	0.025

GAS6-AS1, GAS6-derived long non-coding RNA.

### Animal Experiment

#### Exclusion and Mortality

None of the rats in the sham group died during the present study. Three rats were excluded from the first part of the experiment due to the absence of neurological deficits, and five rats died due to surgical accidents (two of subarachnoid hemorrhage, two of respiratory distress, and one due to an inability to thermoregulate), and eight rats died due to severe neurological deficiencies after surgery (four rats died in each of the HT and rGAS6 groups). One rat was excluded from the second part of our study due to an absence of neurological deficits, while two died of severe neurological deficiency within 24 h and three died of postoperative infections within 36~72 h (two died in the HT group and one in the rGAS6 group). The mortality rates of the two operated groups did not differ significantly.

#### Exogenous rGAS6 Treatment Reduced Brain Infarct Volumes and Hemorrhage Volumes and Improved Neurological Deficits in Hyperglycemia Induced HT Rat Models

**Figure 2:**
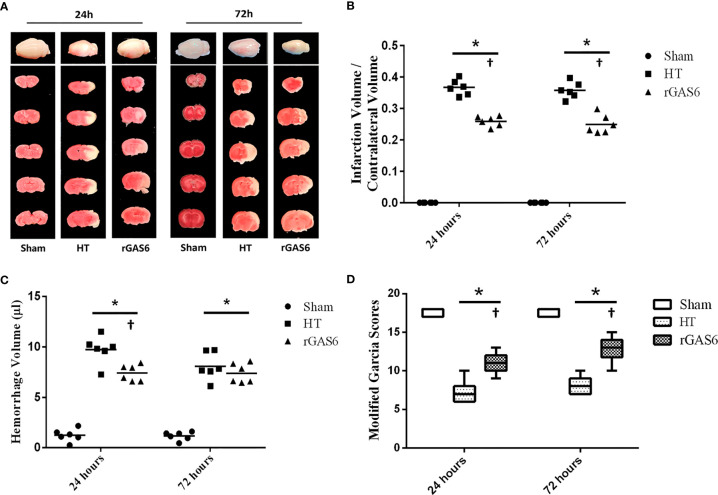
Recombinant GAS6 (rGAS6) treatment reduces infarction and hemorrhagic volume and improves neurological deficits. The whole brain images and slices of rats in the sham, hemorrhagic transformation (HT), and rGAS6 groups stained with 2,3,5-triphenyltetrazolium chloride taken 24 and 72 h after MCAO, respectively, are shown separately in **(A)** (n=6 for each group at each time point). The infarction volume of each group 24 and 72 h after MCAO is shown in **(B)**. The hemorrhagic volume of each group 24 and 72 h after MCAO is shown in **(C)** (n=6 for each group at each time point). The neurologic score of each group 24 and 72 h after MCAO is shown in **(D)** (each group at each time point, n=18). *P < 0.05 versus sham; ^†^ *P* < 0.05 versus hemorrhagic transformation (HT) as shown *via* one-way analysis of variance followed by the Tukey test, or the Kruskal-Wallis test with Bonferroni correction.

We tested brain infarct volumes, hemorrhage volumes, and neurological deficits of rats at 24 and 72 h after MCAO. Infarct volumes in the HT group were significantly increased at both time points compared to those in the sham group. Meanwhile, treatment with rGAS6 significantly reduced infarct volumes compared with those in the HT group (each group at each time point: n=6, *P* < 0.01; **Figures 2A, B**). In addition, hemorrhage volumes in the HT group were significantly higher than those of the sham group at 24 and 72 h after MCAO. rGAS6 treatment significantly reduced the hemorrhage volumes of rats in the HT group 24 h after MCAO (each group, n=6, *P* < 0.01, **Figure 2C**); however, no statistically significant difference was observed at 72 h. Further, hemorrhage volumes in the HT group at 72 h after MCAO tended to be lower than those observed at 24 h, while no apparent differences were found in the rGAS6 group between both time points. Rats in the HT group exhibited significantly reduced neurological function during the neurobehavioral test at 24 and 72 h after MCAO compared to the sham group. Fortunately, the neurological function in the rGAS6 group improved significantly at 24 and 72 h after MCAO compared to that of the sham group (each group at each time point: n=18, *P* < 0.01; **Figure 2D**).

#### Exogenous rGAS6 Treatment Reduced Brain Edema and BBB Permeability in Hyperglycemia Induced HT Rat Models

**Figure 3:**
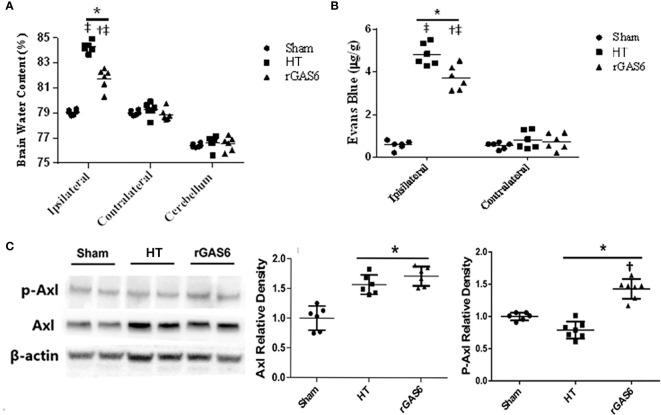
Recombinant GAS6 (rGAS6) treatment reduces brain edema and BBB permeability, as well as enhances Axl phosphorylation (p-Axl). Brain water contents of each group 24 h after MCAO are demonstrated in **(A)** (each group, n=6). Evans blue contents of each group 24 h after MCAO plus 2 h of administration are shown in **(B)** (each group, n=6; 24 h after MCAO). Western blot assays for Axl and p-Axl of each group 24 h after MCAO are shown in **(C)** (each group, n=6). Relative densities of each protein have been normalized to the sham group. *P < 0.05 *versus* sham; ^†^ *P* < 0.05 *versus* hemorrhagic transformation (HT); ^‡^ *P* < 0.05 *versus* contralateral hemisphere, one-way analysis of variance followed by Tukey test.

When compared to the sham group, brain edema in the ipsilateral hemisphere was significantly increased in the HT group, while rGAS6 treatment reduced brain edema in the ipsilateral hemisphere compared with the HT group. Compared with the contralateral hemisphere, brain edema was significantly increased in the ipsilateral hemisphere in the HT group and the rGAS6 group (each group, n=6, *P* < 0.01, **Figure 3A**). However, brain edema in the contralateral hemisphere and cerebellum did not vary significantly between the groups.

In addition, when compared to the sham group, the Evans blue content in the ipsilateral hemisphere was significantly increased in the HT group, while rGAS6 treatment significantly reduced the Evans blue content in the ipsilateral hemispheres. Compared with the contralateral hemisphere, the content of Evans blue in the ipsilateral hemisphere was significantly increased in the HT group and the rGAS6 group 2 h after the 24h MCAO (each group, n=6, *P* < 0.01, **Figure 3B**).

#### Exogenous rGAS6 Treatment Potentiated Axl Phosphorylation in Hyperglycemia Induced HT Rat Models

Western blots demonstrated that the expression of total Axl was significantly increased 24 h after MCAO compared with that in the sham group (each group, n=6, *P* < 0.01, **Figure 3C**). However, rGAS6 treatment did not appreciably alter the expression of total Axl compared with the HT group. Interestingly, the expression of phosphorylated Axl was significantly decreased after MCAO compared with that in the sham group, while rGAS6 treatment significantly increased the expression of phosphorylated Axl compared to the HT group (*P* < 0.01, **Figure 3C**).

## Discussion

In this study, we observed that serum Axl levels were significantly lower in the HT group than in the non-HT group. The abnormally low levels of Axl may be due to gene sequence variations of its related regulatory gene *GAS6-AS1*. Animal experiments further confirmed that Axl agonist treatment (by rGAS6) improves BBB function and reduces HT in hyperglycemia-induced HT rat models by enhancing Axl phosphorylation. These results may assist in identifying AIS patients at higher risk for HT, and also provide potential therapeutic targets for HT treatment.

Axl has been reported to play a key role in maintaining BBB integrity (12, 30). Tong et al. discovered that after using a specific Axl antagonist, brain edema and inflammatory cytokine release were deteriorated in an ICH mouse model (30), suggesting that Axl may be a protective factor for BBB integrity. In the MCAO rat model, Axl activation successfully attenuated neurological dysfunctions by inhibiting the TLR/TRAF/NF-κB inflammatory pathway (31). Since inflammation can promote BBB disruption, it may indicate a possible mechanism for Axl to protect BBB. In our study, we demonstrated that lower levels of Axl were associated with greater opportunities for HT, which also indicated the protective effect of Axl on BBB function. However, it is worth noting that we did not detect an association between *Axl*-associated gene sequence variations and HT following intravenous thrombolysis. The abnormally low levels of Axl could be caused by something other than the *Axl* gene itself.

GAS6 is the ligand of Axl, and previous research indicates that Axl and GAS6 are co-dependent. Axl uniquely depends on GAS6 for activation, and GAS6 requires Axl for stable maintenance *in vivo* (41). This is the reason we detected *GAS6* and *GAS6-AS1* genes after finding low Axl expression in HT patients. Fortunately, we did observe variations in *GAS6-AS1* in HT patients. As the mechanistic investigations found that *GAS6-AS1* can control the expression of the *GAS6* gene at the transcriptional or translational levels by forming a RNA-RNA duplex and promote the Axl signaling activation (28), it is possible that the genetic variation of *GAS6-AS1*, instead of *GAS6*, caused the abnormal expression of *GAS6* at the transcription or translation level, and therefore indirectly caused low Axl expression.

To further investigate the potential correlation between Axl levels and the opportunity for HT, we administered rGAS6 to hyperglycemia-induced HT rats. This model was chosen because hyperglycemia induced HT results in more severe damage to the BBB compared with that induced by tissue plasminogen activator (tPA), allowing for better evaluation of the effect of Axl on BBB function and HT. Our results revealed that rGAS6 treatment significantly potentiated Axl phosphorylation, reduced HT and BBB permeability and improved neurological damages within 24 h after MCAO. We therefore considered Axl a potential effective therapeutic strategy to reduce early HT after stroke.

In addition, we further evaluated the effect of Axl on HT 72 h after MCAO. The results shown that rGAS6 treatment successfully improved neurological deficits and reduced infarct volumes 72 h after MCAO, although it did not affect HT significantly. Meanwhile, hemorrhage volumes in the HT group at 72 h after MCAO tended to be lower than those observed at 24 h, indicating the hematoma clearance in damage sites during 24~72 h after MCAO. However, no apparent difference of hemorrhage volumes were found in the rGAS6 group between both time points. In ICH mouse models, it has been identified that Axl is a pivotal gene in mediating the M2 microglial polarization during the recovery phase of the ICH brain, which promotes the efferocytosis of eryptotic erythrocytes and accelerates hematoma clearance (42). Macrophages significantly upregulated their cell surface expression of Axl both at day 3 and day 7 after ICH. Our experiments suggest a contrary result, that compared with the HT group, rGAS6 treatment might inhibit the hematoma clearance during 24~72 h after MCAO, though no statistical differences of hemorrhage volumes between the two time points were observed in both groups. Whether it suggested a distinctive role of GAS6 in hematoma clearance by regulating underlying mechanisms other than phosphorylating Axl, or if mechanisms accounting for hematoma clearance varied between HT and ICH remains to be found, since infarct damage is more pronounced in HT whereas hemorrhage is induced as a secondary result.

We acknowledge that this study has several limitations. First, the pathway for BBB integrity by GAS6 has not been fully elucidated, though previous study has found the activation of Axl could reduce inflammation and immune response, which may responsible for BBB protection (31). Therefore, additional research is needed to explore the downstream mechanism of Axl in reducing HT and improving BBB function. Second, stroke-induced HT may occur over a longer time, so more studies are needed to investigate the effect of rGAS6 on delayed HT. Third, as we chose to use the hyperglycemia-induced HT model, which can result in more severe damage to the BBB compared to the tPA-induced HT model. However, the downside of this choice is that animal and human experiments are not consistent, leaving us a still question whether rGAS6 can provide a similar protective effect in tPA induced HT. Finally, the sample size of our clinical study is relatively small. These findings need to be further investigated in large-scale studies.

## Conclusions

The present study suggests that lower serum Axl levels, which may result from sequence variations of *GAS6-AS1*, are associated with an increased risk of HT after intravenous thrombolysis in AIS patients. Activation of the Axl signaling pathway by the GAS6 protein may serve as a therapeutic strategy to reduce HT in AIS patients.

## Data Availability Statement

The raw data supporting the conclusions of this article will be made available by the authors, without undue reservation.

## Ethics Statement

The studies involving human participants were reviewed and approved by the Ethics Committee of the First Hospital of Jilin University. The patients/participants provided their written informed consent to participate in this study. The animal study was reviewed and approved by the Experimental Animals Ethics Committee of Jilin University of China.

## Author Contributions

YY and Z-NG drafted the initial protocol, which was reviewed with critical revisions and approval by all authors. PZ did the statistical analysis. YY, Z-NG, and JL wrote the first draft of the manuscript. All authors contributed to data acquisition. All authors contributed to critical revision of the manuscript and approved the final manuscript for submission.

## Funding

This work was supported by the National Natural Science Foundation of China to YY (Grant No. 81771243), the Program for JLU Science and Technology Innovative Research Team (2017TD-12) and Jilin Provincial Key Laboratory (20190901005JC) to YY.

## Conflict of Interest

The authors declare that the research was conducted in the absence of any commercial or financial relationships that could be construed as a potential conflict of interest.

## Publisher’s Note

All claims expressed in this article are solely those of the authors and do not necessarily represent those of their affiliated organizations, or those of the publisher, the editors and the reviewers. Any product that may be evaluated in this article, or claim that may be made by its manufacturer, is not guaranteed or endorsed by the publisher.
